# Progress in the Fabrication and Optimization of High-Energy Diamond X-Ray Refractive Lenses

**DOI:** 10.3390/mi17060687

**Published:** 2026-06-01

**Authors:** Hao Huang, Kang Du, Wenbin He, Weiwei Zhang, Xiaohong Yang, Wuyi Ming

**Affiliations:** 1 Fair Friend Institute of Intelligent Manufacturing, Hangzhou Polytechnic University, Hangzhou 310018, China; 2024010031@hzvtc.edu.cn; 2 Henan Provincial Key Laboratory of Intelligent Manufacturing of High-End Equipment, Henan Province Engineering Technology Research Center of Green Manufacturing and Precision Measurement, Zhengzhou University of Light Industry, Zhengzhou 450002, China; 18438278147@163.com (K.D.); hwb@zzuli.edu.cn (W.H.); 3 Institute of High Energy Physics, Chinese Academy of Sciences, 19B Yuquan Road, Shijingshan District, Beijing 100049, China; 4 Hebi Institute of Engineering and Technology, Henan Polytechnic University, Hebi 458030, China; yangxiaohong@hpu.edu.cn; 5 Guangdong HUST Industrial Technology Research Institute, Huazhong University of Science and Technology, Dongguan 523808, China

**Keywords:** Diamond, X-ray refractive lenses, Synchrotron radiation, XFEL, micro/nanofabrication, surface quality, thermal management

## Abstract

The extreme thermal loads encountered in fourth-generation synchrotron radiation sources and X-ray free-electron lasers (XFEL) impose stringent requirements on X-ray optical components. Conventional materials such as beryllium and silicon increasingly exhibit limitations under high-energy conditions, including insufficient thermal conductivity, limited radiation stability, and significant absorption losses, rendering them inadequate for next-generation high-energy X-ray optics. In this context, single-crystal diamond, with its high thermal conductivity, low absorption coefficient, and excellent mechanical strength and radiation resistance, has emerged as a promising candidate for high-energy X-ray refractive optics. This review systematically summarizes recent advances in the fabrication and performance optimization of diamond X-ray refractive lenses for high-energy applications. Starting from the evolving demands of modern synchrotron radiation facilities and XFEL, the fundamental requirements for materials and structural design in high-energy X-ray optics are analyzed. Through comparisons with representative materials, the advantages of diamond in thermal management and transmission performance are highlighted. Major micro- and nanofabrication techniques, including femtosecond laser processing, focused ion beam milling, and plasma etching, are comprehensively reviewed, with emphasis on their respective characteristics in terms of processing efficiency, precision control, and damage introduction. The emerging trend of hybrid fabrication strategies is also discussed. Furthermore, the effects of surface roughness, subsurface damage, and crystal defects on wavefront quality and focusing performance are examined, along with corresponding post-processing and surface correction methods. Finally, current challenges related to large-size single-crystal growth, high-precision low-damage fabrication, and long-term operational stability are discussed, and future development directions for diamond-based X-ray refractive optical components are outlined.

## 1. Introduction

**Figure 1:**
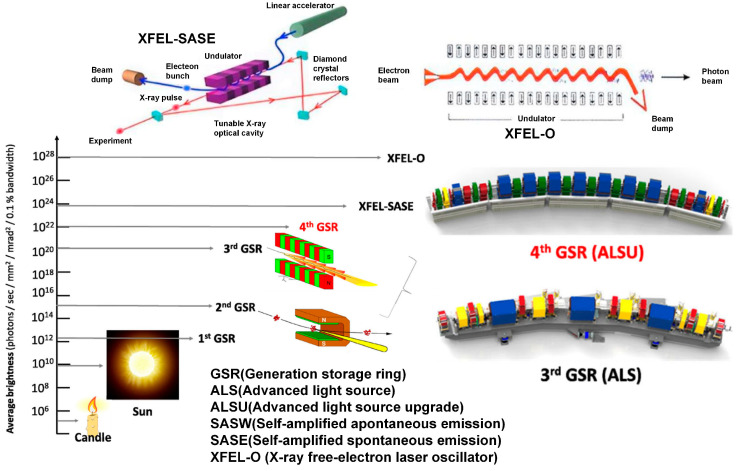
Evolution of average luminosity from a candle to an XFEL [1].

Synchrotron radiation sources, characterized by extremely high brightness, excellent collimation, a continuously tunable broad energy spectrum, and steadily improving spatial coherence, have become indispensable facilities for modern precision scientific research. The development of synchrotron radiation technology has undergone a continuous evolution from parasitic sources to dedicated storage rings and, more recently, to low-emittance storage rings. Current fourth-generation synchrotron light sources [1], based on diffraction-limited storage ring designs, reduce the electron beam emittance to near the diffraction limit. Compared with third-generation sources, the X-ray brightness and coherent photon flux have increased by several orders of magnitude, significantly expanding experimental capabilities in coherent scattering, coherent imaging, and nanoscale structural characterization [2,3]. Figure 1 illustrates the exponential growth in X-ray brilliance and coherent photon flux from the first to the fourth generation of synchrotron radiation sources. With the development of fourth-generation sources and X-ray free-electron lasers (XFEL), the ultra-high brilliance and high coherence of these beams have imposed more stringent requirements on the thermal stability, radiation hardness, and mechanical reliability of X-ray optical components. Synchrotron radiation has played a central role in a wide range of frontier fields, including materials science, life sciences, chemistry, microelectronics, and condensed matter physics [4]. Emerging coherent X-ray sources, typified by XFEL, have achieved ultra-high peak brilliance and ultra-short pulses with near-complete coherence. These capabilities establish a novel experimental paradigm for probing ultrafast dynamics and transient structural evolution at the atomic scale [5]. As synchrotron facilities continue to advance toward higher photon energies, higher power densities, and greater coherence, the thermal and radiation loads imposed on beamline X-ray optical systems have increased substantially [6]. Under fourth-generation source conditions, high-flux X-ray beams can induce pronounced thermal deformation and stress concentration in optical components, thereby compromising beam stability and ultimate experimental resolution [7]. Consequently, modern synchrotron X-ray optics are required not only to achieve high reflectivity or transmission efficiency, but also to maintain excellent thermal stability, structural integrity, and sub-micrometer to nanometer-scale optical precision under intense irradiation and high heat flux conditions [8]. These stringent requirements have directly driven the development of optical materials with high thermal conductivity, low thermal expansion, and superior radiation tolerance, as well as innovative X-ray optical component architectures, forming a key enabling direction for next-generation synchrotron radiation technologies [9].

**Figure 2:**
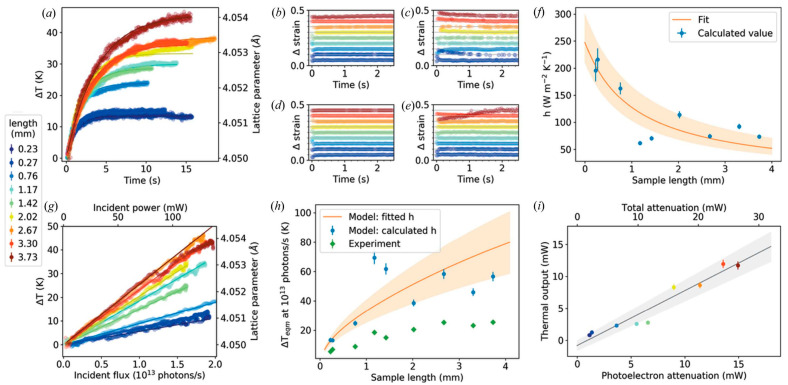
Transient thermal response and thermally induced strain evolution of an aluminum wire under high-flux X-ray irradiation [7]. (**a**) Transient temperature evolution following beam insertion; (**b**–**e**) Diffraction strains of the (113), (222), (133), and (024) lattice planes; (**f**) Relationship between the heat transfer coefficient h and sample length; (**g**) Temperature response as a function of incident photon flux; (**h**) Comparison between experimental measurements and model predictions for temperature variations at 10^13^ photons/s; (**i**) Calculated thermal output versus X-ray beam attenuation.

The development of high-energy X-ray optics is confronted with a series of fundamental challenges arising from harsh service conditions. Insufficient thermal conductivity or inadequate heat dissipation in optical components often induces severe temperature gradients and thermal deformation. These effects degrade optical performance and beam stability [7,10]. As illustrated in Figure 2, Lawrence et al. [7] systematically investigated the transient thermal response and strain evolution of a 0.5 mm diameter aluminum wire model sample subjected to high-flux X-ray irradiation. Figure 2a depicts the temporal evolution of the sample temperature following the insertion of a focusing lens into the beam path. This result demonstrates a rapid temperature surge toward a new thermal equilibrium under high photon flux. Figure 2b–e present the evolution of diffraction strains for the (113), (222), (133), and (024) crystallographic planes in response to thermal loading. These thermal loads induce dynamic variations in lattice parameters and consequently compromise X-ray wavefront stability and diffraction quality. In XFEL-based highly coherent imaging and nanofocusing systems, such thermally induced wavefront distortions can lead to focal spot broadening, diminished peak intensity, and degraded imaging resolution. Figure 2f reveals the coupling relationship between the heat transfer coefficient h and the sample length, which highlights the profound impact of geometric dimensions on thermal diffusion capabilities. In addition, Figure 2g exhibits an approximately linear correlation between the temperature increment and the incident photon flux. This trend implies that the challenges of thermal management will escalate rapidly with further enhancements in XFEL peak power. Figure 2h compares the experimental measurements with the predictions of a thermal model and validates the efficacy of the thermal transport model under high-flux X-ray conditions. Figure 2i calculates the variation in thermal output as a function of X-ray attenuation at a flux of 10^13^ photons/s. This calculation provides critical insights for the thermal design and material selection of XFEL optical systems. This study elucidates the mechanisms by which thermo-structural coupling affects optical stability under high-brilliance XFEL irradiation and underscores the necessity of developing optical materials with high thermal conductivity, low thermal expansion, and robust radiation resistance. The achievable spatial resolution of X-ray focusing and imaging techniques is strongly dependent on the fabrication accuracy and surface quality of optical elements. Even micrometer- or nanometer-scale form errors and surface roughness can significantly deteriorate wavefront quality and focusing efficiency [11,12,13]. The combination of high-energy radiation environments with stringent fabrication and long-term operational requirements imposes exceptionally high demands on the mechanical strength, radiation tolerance, and chemical stability of optical materials [8,14].

As the photon flux of fourth-generation synchrotron radiation sources and XFEL continues to increase, X-ray optical components face material degradation caused by long-term high-energy radiation in addition to extreme thermal loads [1,15,16]. Continuous exposure to high-energy X-rays can induce radiation damage within optical materials, such as point defects, lattice distortion, and localized amorphization. These processes result in decreased thermal conductivity, degraded crystal integrity, and reduced wavefront stability [17,18]. In highly coherent X-ray optical systems, even minimal lattice defects or localized strains can cause phase distortion and enhanced scattering [19]. These effects lead to reduced focusing performance and negatively impact the spatial resolution and measurement stability of nanoscale experiments [18,20]. Conventional optical materials and device designs often fail to simultaneously satisfy multiple constraints including thermal management, fabrication precision, radiation stability, and long-term service reliability [8,13]. This limitation has become a critical bottleneck for the further development of high-energy X-ray optical systems.

To overcome multiple material and structural constraints under extreme service environments, extensive interdisciplinary explorations have been conducted in materials science and advanced manufacturing in recent years [21]. Diamond effectively fulfills the specific requirements of high-energy X-ray optics for high transmission and high coherence owing to its superior thermophysical and optical properties [22,23,24]. Under conditions of ultra-high flux or long-term irradiation, radiation-induced defects, residual stress accumulation, and localized thermal damage can still occur in single-crystal diamond (SCD). These phenomena compromise thermal transport capacity and optical stability [24,25]. The progression of high-energy X-ray optics toward higher resolution and coherence imposes stricter requirements on the surface integrity, fabrication precision, and manufacturing defect control of diamond optical components [22]. Achieving high-quality fabrication, surface damage suppression, and improved long-term radiation stability for SCD optical elements has become a primary research focus in high-energy X-ray optics.

**Figure 3:**
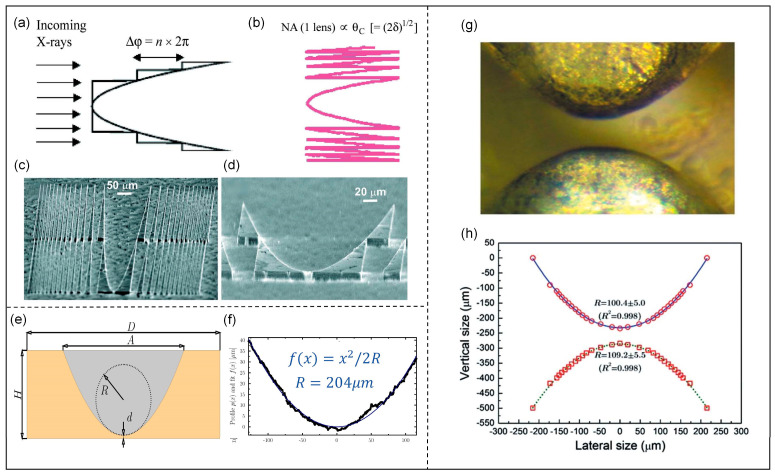
Diamond X-ray refractive lens configurations and working principles. (**a**) Long kinoform lens [29]; (**b**) Short kinoform lens [29]; (**c**) Diamond kinoform lens for focused imaging at E = 11.3 keV and f = 0.2 m [29]; (**d**) Side view of the central region of a 7.4 keV lens from the incident X-ray perspective [29]; (**e**) Schematic diagram of a parabolic lens [30]; (**f**) Centerline profile of the diamond lens surface topography [30]; (**g**) View of a double-concave two-dimensional parabolic lens [31]; (**h**) Profile of a double-concave two-dimensional parabolic lens and the parabolic fit based on the radius of curvature [31].

X-ray refractive lenses achieve beam focusing primarily by utilizing the extremely small refractive index decrement in materials [26]. The weak refractive effect of X-rays necessitates the use of rotationally symmetric or two-dimensional parabolic geometries to minimize spherical aberration and enhance focusing efficiency [27,28]. Diamond-based X-ray refractive optics currently employed in synchrotron radiation and X-ray free-electron laser systems include compound refractive lenses (CRL), planar or rotationally symmetric parabolic lenses, kinoform lenses, and phase-type focusing devices based on micro-nanostructure modulation [29,30,31]. CRL effectively enhances focusing power by arranging multiple parabolic lens units in series. In contrast, kinoform and phase-type structures optimize wavefront modulation performance while simultaneously reducing absorption losses. Different lens types exhibit distinct variations in their focusing mechanisms, fabrication processes, and wavefront control capabilities. Figure 3 illustrates the structural forms and fundamental focusing principles of typical diamond X-ray refractive lenses. Diamond refractive optics are considered a critical development direction for next-generation high-coherence X-ray beamlines due to their low X-ray absorption, high thermal conductivity, excellent radiation resistance, and superior wavefront preservation capabilities [22,32]. This review aims to provide a comprehensive overview of recent advances in diamond-based X-ray refractive optics, with particular emphasis on fabrication technologies, surface quality control, and optical performance optimization strategies under harsh synchrotron radiation conditions.

## 2. Application Requirements and Material Suitability

High-energy X-ray refractive optical systems are key components in modern light sources for achieving nanoscale spatial resolution and ultrahigh photon flux density. With the continuous increase in photon energy and beam brightness, these optical elements are subjected to increasingly stringent physical and engineering requirements. To maximize transmission efficiency while minimizing wavefront distortion, lens materials must exhibit exceptional crystal purity, extremely low X-ray absorption, and superior thermal and mechanical stability.

### 2.1. Synchrotron and XFEL Requirements

The continuous evolution of advanced X-ray sources imposes increasingly stringent requirements on high-performance X-ray optical systems. Early generation synchrotron radiation (GSR) facilities primarily operated as parasitic sources for high-energy physics accelerators. Their limited brilliance and spatial coherence restricted X-ray optical systems mainly to beam transport and basic focusing tasks [1]. The transition to third-generation synchrotrons and advanced light sources (ALS) brought significant improvements in brilliance, collimation, and spatial coherence. This shift enabled the rapid development of high-resolution imaging, coherent diffraction, and micro-nanofocusing techniques. Consequently, wavefront preservation, thermal stability, and surface topography precision became essential metrics for evaluating optical performance [33,34].

The introduction of diffraction-limited storage ring upgrades, such as the Advanced light source upgrade (ALS-U), and self-amplified spontaneous emission X-ray free-electron lasers (SASE-XFEL) pushed X-ray science toward ultra-high brilliance, high coherence, and femtosecond time resolution [1,35]. Facilities like the linac coherent light source [36] and the European XFEL [37] generate femtosecond X-ray pulses with ultra-high peak brilliance. These pulses provide vital platforms for research in ultrafast pump–probe studies, femtosecond crystallography, and coherent diffraction imaging.

With the advancement of XFEL oscillators (XFEL-O) and seeded XFEL systems, light sources now target higher temporal coherence, lower noise, and more stable pulse outputs [38,39]. This progress necessitates that X-ray optical systems exhibit high transmittance and low absorption while maintaining superior wavefront fidelity and thermal stability under extreme radiation. Next-generation materials must provide a combination of low X-ray absorption, high thermal conductivity, low thermal expansion, and excellent radiation resistance [11,40]. Table 1 summarizes the beam characteristics and optical requirements of typical light sources to track the evolution of these performance demands.

Early high-energy X-ray focusing systems relied primarily on reflective optics, diffractive optics, and CRL. Low-atomic-number materials such as beryllium were widely adopted in these systems due to their low X-ray absorption [41]. Research indicates that these conventional materials gradually exhibit limitations under high-brilliance synchrotron and XFEL conditions. These limitations include limited thermal conductivity, insufficient radiation resistance, and significant thermal deformation [26,42]. Some traditional inorganic materials still suffer from absorption losses in the hard X-ray energy range. These losses reduce system transmission efficiency and wavefront stability [32,43]. The study of advanced diamond X-ray optical devices represents a major development direction for fourth-generation synchrotron radiation and XFEL sources.

### 2.2. Advantages of Diamond Optics

SCD has demonstrated great potential for high-energy X-ray optics, exhibiting significant advantages over conventional materials such as beryllium and silicon [44]. With a thermal conductivity of up to about 2000 W/(m·K) at room temperature, diamond provides exceptional heat dissipation capability under high-power synchrotron radiation or XFEL irradiation, effectively suppressing thermal deformation and thermal lensing effects [45]. In addition, SCD possesses extremely high hardness and elastic modulus, along with outstanding structural stability and radiation tolerance, enabling reliable long-term operation under high heat flux densities and complex service environments [46]. Its X-ray absorption coefficient is substantially lower than that of most metallic materials, resulting in high transmission and reduced background scattering in the hard X-ray regime, which is beneficial for improving focusing efficiency and imaging contrast. In recent years, the design and experimental validation of CRL and axicons have achieved significant progress, demonstrating their high-energy focusing capability and thermal stability in fourth-generation synchrotron facilities [47]. Hedayat et al. [48] successfully addressed the constraints of conventional materials such as Be and Si concerning thermal load and absorption losses. Zholudev et al. [49] validated the superior heat dissipation capabilities of diamond that effectively suppress thermal distortion and thermal caustic effects. Table 2 summarizes the physical properties of various materials. Diamond has emerged as one of the most competitive candidates for high-energy X-ray refractive optical systems.

### 2.3. Early Research Developments

**Figure 4:**
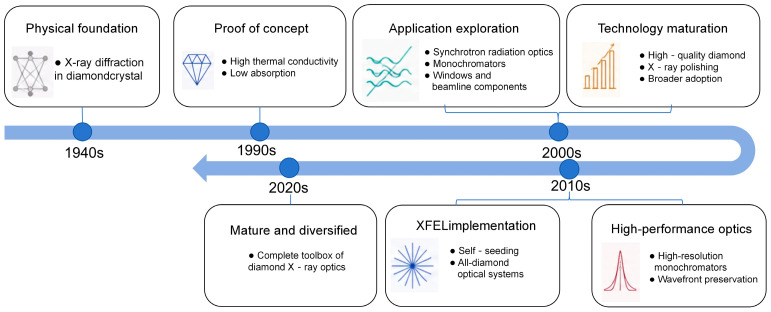
Evolution of diamond X-ray optics.

Research on diamond in the field of X-ray optics has followed a progressive evolution from fundamental physical verification to the systematic development of high-end optical devices, as illustrated in Figure 4. Early studies date back to the mid-20th century during the exploration of crystal X-ray diffraction physics. For instance, Raman et al. [56] investigated X-ray diffraction and energy exchange phenomena in diamond crystals. This work established the physical foundation for the subsequent use of crystalline materials in X-ray modulation. With the emergence of third-generation synchrotron radiation sources, the demand for X-ray optics capable of handling high heat loads increased rapidly. Berman et al. [57] first systematically verified the feasibility of single-crystal diamond as a high-power-density X-ray monochromator. Their work demonstrated that diamond maintains minimal thermal distortion and excellent wavefront preservation under extreme radiation conditions. This milestone marked the transition of diamond from a subject of basic crystal research to an engineered X-ray optical material. Subsequent research shifted from feasibility verification to the expansion of applications. Diamond was extensively studied for use in synchrotron monochromators, windows, and high-heat-load optical components. These efforts confirmed its advantages in thermal conductivity, radiation stability, and low absorption [58,59]. Around 2010, Shvyd’ko et al. [23] achieved significant breakthroughs in high-resolution crystal optics. They demonstrated that diamond provides extremely high reflectivity and superior energy resolution under Bragg diffraction. This capability expanded its application into the scope of high-coherence X-ray optics.

Following the development of XFEL, diamond optical components were incorporated into practical beamline systems for self-seeding monochromatization and high-stability beam control. These implementations highlighted the unique advantages of diamond under extreme peak power and ultra-short pulse conditions [60,61]. In recent years, research in this field has become more systematic and has produced a comprehensive range of devices. These include monochromators, beam splitters, phase retarders, and high-precision windows. Diamond has since become an indispensable core X-ray optical material for advanced synchrotron and XFEL facilities [22]. Related studies have also shown that single-crystal diamond in channel-cut, single-crystal, and double-crystal configurations can function as highly stable monochromators [46]. Thin diamond plates have also been employed as transmissive beam splitters in synchrotron and XFEL experiments [55,62]. These works highlight the advantages of diamond in material processing, wavefront preservation, and thermal management [22,46].

With the growing understanding of the X-ray optical properties of diamond, research has progressively expanded to its application in X-ray focusing devices [26,63]. Owing to their low X-ray absorption and exceptional thermal stability, CRL have been demonstrated to be well suited for efficient focusing of hard X-ray beams [31,64]. Both theoretical and experimental studies indicate that, compared with other materials, diamond CRL can maintain superior wavefront fidelity and reduced diffraction losses under high radiation power, making it a promising solution for high-brightness X-ray imaging and focusing systems [44,65].

The exceptional physical properties of diamond also impose stringent challenges on high-precision three-dimensional surface fabrication. As early as 2016, Kononenko et al. [66] overcame the limitations of conventional techniques in terms of etching depth and surface quality, demonstrating the feasibility of fabricating compound refractive X-ray lenses directly in polycrystalline chemically vapor-deposited (CVD) diamond plates up to 600 μm thick using femtosecond laser ablation. By exploiting the “cold ablation” characteristics of ultrashort pulses, submicrometer sidewall roughness was achieved, and an effective focal length of 97 cm was experimentally realized at 9.25 keV, establishing femtosecond laser processing as a key technique for diamond micro/nanofabrication. A 2019 study revealed that CRL can induce spectral dips in synchrotron applications due to crystal orientation and localized Bragg diffraction. This result indicates that crystal integrity, orientation precision, and defect control directly influence wavefront stability and optical performance [67]. In 2020, researchers utilized ion beam lithography to fabricate high-precision diamond microlenses. By optimizing the micro-nanofabrication process, they significantly reduced sidewall profile errors and surface roughness. This approach achieved X-ray focusing performance that approaches the diffraction limit [68]. In recent years, significant progress has been achieved in the fabrication of diamond X-ray refractive lens structures using advanced micro/nanomanufacturing techniques, particularly femtosecond laser ablation [45]. Lens arrays incorporating aberration compensation mechanisms have demonstrated extremely small focal spot sizes across both soft and hard X-ray regimes. For example, Wang et al. [69] achieved a focal spot of approximately 52 nm × 51 nm at 14 keV, highlighting the potential of such devices for application in fourth-generation synchrotron and XFEL beamlines. Diamond refractive optical elements exhibit excellent wavefront preservation and low diffraction losses under high-brightness beams, making them particularly suitable for high-resolution applications.

To address residual form errors and surface roughness that are difficult to eliminate during primary fabrication, focused ion beam (FIB) nanomachining provides an ultimate solution. Through chip-level precision aberration correction on the lens surface, FIB-based finishing effectively removes residual wavefront distortions, enabling focal spot sizes on the order of about 50 × 60 nm^2^ [70]. The integration of ultrafast laser-based macroscopic shaping with FIB-enabled nanoscale surface refinement offers a promising pathway toward the development of low-aberration, high-performance diamond X-ray optical components.

## 3. Fabrication Strategies of Diamond X-Ray Refractive Lenses

Due to its status as the hardest known natural material and its exceptional chemical inertness [44], diamond is extremely difficult to process using conventional techniques. Traditional mechanical methods, such as turning and grinding, as well as standard wet chemical etching, are incapable of achieving precise micro-/nanoscale three-dimensional surface control on diamond substrates [71]. High-energy X-ray refractive lenses, particularly CRL, typically require high-aspect-ratio parabolic or cylindrical geometries, along with stringent requirements on surface roughness and form accuracy. The development of micro/nanofabrication techniques that simultaneously ensure high precision and minimal subsurface damage has therefore become a key enabling factor for translating diamond X-ray lenses from theoretical designs to practical applications. At present, mainstream fabrication approaches are primarily based on advanced processing techniques, including femtosecond laser ablation [72], FIB milling [73], and reactive ion etching (RIE) [74].

### 3.1. Femtosecond Laser Processing

**Figure 5:**
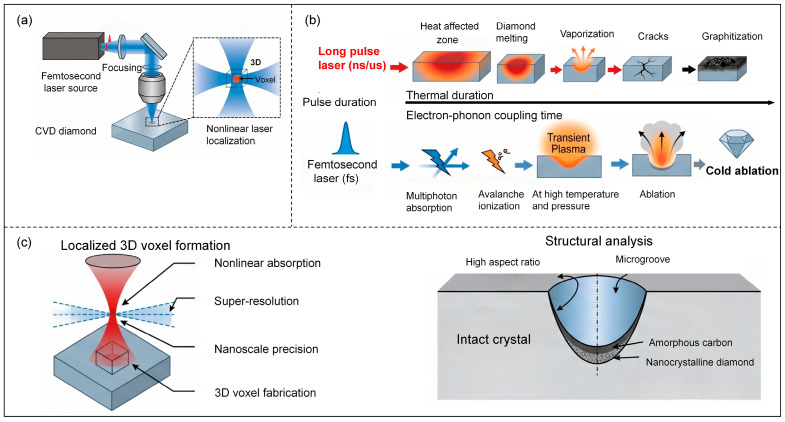
Schematic of the femtosecond laser system and its working principle. (**a**) System configuration and laser–material interaction; (**b**) Micromachining mechanisms; (**c**) Resulting morphology.

Among existing fabrication methods for diamond X-ray optics, femtosecond laser technology represents one of the most promising approaches owing to its unique physical mechanisms. Figure 5 illustrates the system setup for the femtosecond laser processing of CVD diamond. This figure compares ablation mechanisms across different pulse durations and analyzes the resulting microstructural characteristics. The ultra-short pulse durations of 30 to 300 fs and peak power levels as high as 5 × 10^20^ W/m^2^ provide high flexibility and relatively high production efficiency for three-dimensional micro-nanofabrication [51].

In terms of material removal mechanisms, femtosecond laser ablation effectively overcomes the machining damage challenges associated with transparent ultra-hard materials. Liu et al. [75], in their study on hybrid laser precision manufacturing of transparent hard materials, pointed out that diamond and similar materials exhibit exceptionally high transmittance and very low absorption under conventional long-pulse laser irradiation. In contrast, femtosecond pulses, owing to their extremely high instantaneous peak power, can induce strong nonlinear multiphoton absorption, leading to transient plasma formation and subsequent material removal via vaporization and ejection. The pulse duration of femtosecond lasers is significantly shorter than the electron–phonon coupling time within the diamond lattice, as shown in Figure 5b. This “cold ablation” mechanism enables material removal before thermal energy diffuses into the surrounding lattice and restricts the heat-affected zone to a minimal range. This process avoids thermally induced microcracks and the graphitization of the diamond, which preserves the thermal stability and intrinsic crystal quality of the component.

**Figure 6:**
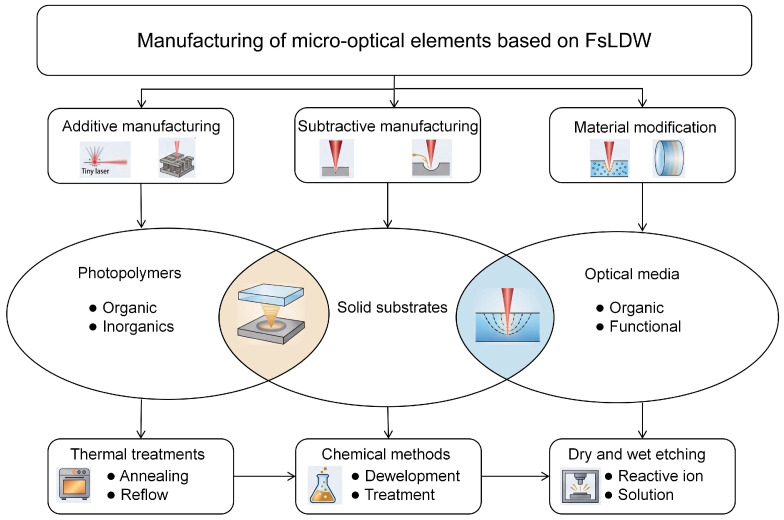
Femtosecond laser direct writing for the fabrication of optical devices.

For complex three-dimensional microstructures shown in Figure 5c, femtosecond lasers can create localized 3D voxel structures within the material. This fabrication process relies on high peak power and nonlinear multiphoton absorption to achieve confined energy deposition and sub-diffraction-scale machining. As illustrated in Figure 6, femtosecond laser direct writing (FsLDW) has been used to fabricate various micro-optical elements. This technique demonstrates high-degree-of-freedom 3D manufacturing capabilities that are difficult to achieve with conventional lithography. Huang et al. [76]. detailed how FsLDW exploits the threshold effect of nonlinear absorption to confine energy deposition within an extremely small three-dimensional voxel at the focal point. This highly localized interaction enables processing resolutions beyond the optical diffraction limit, achieving voxel sizes below 100 nm in transparent materials and spatial resolutions down to 10 nm in specific systems such as POSS glass. As a true three-dimensional maskless fabrication technique, FsLDW can directly “sculpt” high-aspect-ratio parabolic or complex phase-type structures in diamond with nanometer-level form precision, without the need for lithographic masks or etching templates. Ali et al. [51] systematically reviewed the differences in material removal mechanisms between femtosecond and long-pulse lasers. Long-pulse laser processing is primarily governed by thermal melting and evaporation, whereas femtosecond lasers, under high-intensity focusing conditions, induce strong nonlinear absorption through multiphoton ionization and avalanche ionization. This leads to ultrafast energy deposition into the electronic system and the formation of a high-temperature, high-pressure plasma within sub-picosecond timescales, enabling transient material removal.

During laser processing of CVD diamond with 216 fs pulses, the damage threshold is strongly influenced by both wavelength and incubation effects. Nolasco et al. [77] utilized a femtosecond laser system with a repetition rate of 100 Hz–1 MHz to investigate the effects of different wavelengths on the ablation behavior of single-crystal diamond at a pulse width of 216 fs. The results demonstrated that the single-pulse ablation thresholds for the 1030 nm, 515 nm, and 343 nm lasers were approximately 1–2 J/cm^2^. Under multi-pulse irradiation, the ablation threshold further decreased to a range of 0.03–0.20 J/cm^2^ due to defect accumulation driven by the incubation effect. Zou et al. [78] employed a Yb:KGW femtosecond laser system with a 190 fs pulse width and a 200 kHz repetition rate to perform micro-trench etching on single-crystal diamond. Their results indicated that the 513 nm laser yielded a stable trench width of approximately 2 μm, whereas the 1026 nm laser increased the trench width to about 4 μm at higher energy densities. By utilizing multiple scanning passes at a low energy density, a trench depth of approximately 2–3 μm could be achieved while maintaining a well-defined edge morphology. Transmission electron microscopy analysis further revealed that the sidewall regions not directly exposed to intense laser irradiation exhibited minimal damage, with an interplanar spacing of 0.206 nm, indicating preservation of the crystalline structure. In contrast, the groove bottom formed amorphous carbon and nanocrystalline diamond phases due to localized energy concentration. This process enables the fabrication of high-quality grid-like structures with controllable bottom roughness, making it suitable for precision micro/nanostructuring of diamond surfaces. The “cold ablation” characteristics of femtosecond laser processing effectively suppress thermal diffusion and graphitization, providing significant advantages for the fabrication of deep, high-precision parabolic lens profiles inside or on the surface of diamond while maintaining low surface roughness and excellent structural integrity [79].

### 3.2. Ion Beam Machining

**Figure 7:**
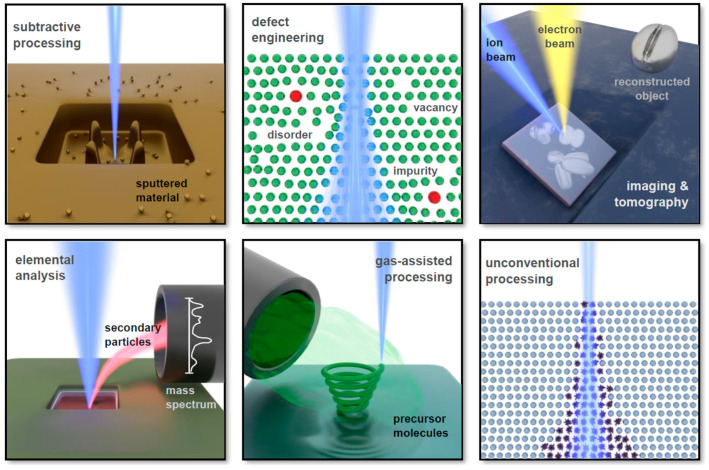
Schematic overview of different FIB techniques [80].

In the field of high-precision surface machining at the micro- and submicron scale, FIB technology has demonstrated irreplaceable advantages [80,81]. Figure 7 illustrates different types of FIB techniques. Under conventional high-resolution operating conditions, FIB processing can achieve nanometer-scale spatial resolution.

The material removal mechanism of this technique is primarily based on physical sputtering induced by momentum transfer between high-energy incident ions and target atoms in the solid. Shin et al. [82] conducted a systematic study on the processing of 80 μm thick diamond using a 30 keV plasma-focused ion beam. The results show that introducing an oxygen (O_2_) ion beam enables chemically assisted etching via the formation of CO and CO_2_, effectively overcoming the efficiency limitations of pure physical sputtering in high-aspect-ratio deep structures. Meanwhile, the sidewall taper angle of the fabricated microstructures can be precisely controlled at approximately 5°. The sidewalls produced by the O_2_-PFIB process exhibit the smoothest surface quality. This capability, combining bulk material removal with nanoscale roughness control, makes PFIB a key enabling technique for fabricating high-aspect-ratio diamond X-ray refractive lenses under stringent requirements. As summarized by Höflich et al. [80], the material removal mechanism of FIB is mainly governed by physical sputtering caused by momentum transfer from high-energy gallium ions to target atoms. Under typical high-precision conditions, the spatial resolution can reach a full width at half maximum of approximately 2–2.8 nm. During momentum transfer, collision cascades are also generated within the material. For example, 30 keV Ga^+^ ions can have a maximum penetration depth of ~60 nm, which may introduce surface amorphization and ion implantation damage when processing sensitive materials such as diamond-based quantum devices. Medvedskaya et al. [68] investigated ion beam lithography for fabricating rotational parabolic diamond micro-lenses for high-resolution X-ray imaging. They demonstrated that, compared with laser ablation, FIB enables smooth, speckle-free structures with extremely small radii of curvature, meeting the stringent surface roughness requirements of ultraminiaturized X-ray optics. According to the ion beam shaping experiments reported by Shurvinton et al. [83], the material removal rate of ion beams on optical components is on the order of about 1 nm/s. By precisely controlling dwell time, sub-nanometer removal accuracy can be achieved. The figure error of X-ray optical surfaces can be reduced from an initial 8 nm RMS to 0.76 nm RMS (and in some cases down to 0.36 nm RMS), while the tangential slope error can be decreased from 411 nrad RMS to 97 nrad RMS.

Conventional FIB techniques still face two major limitations in practical applications: (1) the material removal rate is extremely low due to the limited beam current of liquid metal ion sources, making it unsuitable for bulk machining of large-aperture lenses [84]; and (2) high-energy ion implantation can induce significant subsurface damage [85]. Burnett et al. [86] pointed out that when attempting to increase the beam current of a Ga^+^ liquid metal ion source beyond 5 nA, severe spherical aberration dominates the beam profile, resulting in a non-Gaussian distribution with pronounced tails, thereby preventing simultaneous high-current operation and high-resolution focusing. Consequently, the machining depth and volume of conventional Ga^+^ FIB are typically limited to the order of tens of micrometers, making it unsuitable for large-volume material removal in bulk components. During processing, high-energy Ga^+^ ions not only induce collision cascades and physical damage but also, due to their chemical reactivity, may trigger chemical interactions or phase transformations within the material. The use of inert gas xenon ion (Xe^+^) PFIB can effectively mitigate these physical and chemical damages while overcoming aberration limitations at high beam currents, achieving material removal rates approximately 60 times higher than those of conventional Ga^+^ FIB. Rubanov et al. [87] revealed the damage limitations of 30 keV Ga^+^ FIB in diamond micro/nanofabrication: ion implantation disrupts the diamond lattice, forming an amorphous damaged layer up to ~44 nm deep near the surface. This layer contains both sp^2^ and sp^3^ bonding configurations, exhibiting amorphous characteristics. The structural damage reduces the material density to 2.25 g/cm^3^ and induces volume expansion, resulting in local surface swelling of up to 13 nm. Glushkov et al. [88] confirmed that high-energy ion bombardment generates point defects and localized amorphization. When such ion-induced amorphous regions are exposed to aqueous environments, water-assisted etching can occur, selectively removing the damaged material and leading to pronounced morphological and optical changes in the affected regions.

### 3.3. Plasma Etching

RIE has recently made significant progress in the field of diamond micro/nanofabrication, extending from fundamental innovations in etching mechanisms to breakthroughs in hard-mask engineering. The introduction of RIE and inductively coupled plasma reactive ion etching (ICP-RIE) enables large-scale and highly uniform fabrication of diamond X-ray refractive optical elements with excellent reproducibility.

**Figure 8:**
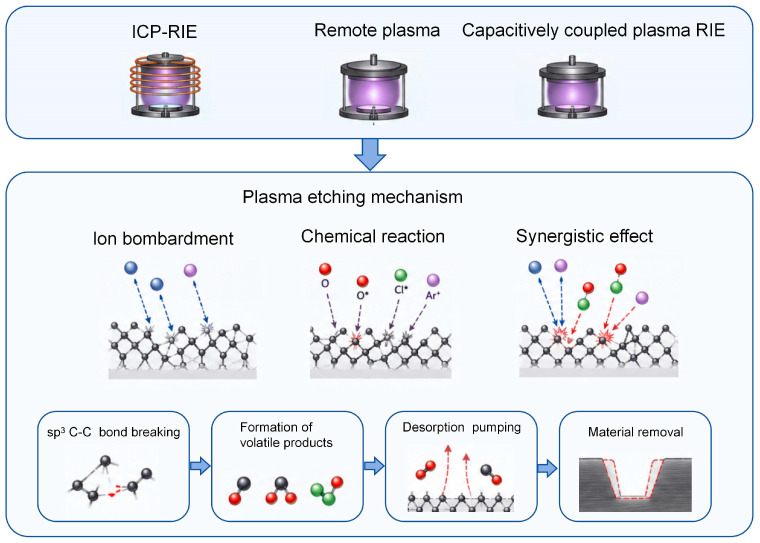
Plasma-assisted etching of diamond for X-ray optical structures.

Compared with laser- and ion-beam-based techniques, plasma etching offers distinct advantages in achieving large-area, uniform, and repeatable pattern transfer, which is particularly beneficial for the fabrication of lens arrays and other planar optical architectures. In ICP-RIE processes, material removal results from the synergistic interaction between physical ion bombardment and chemically assisted surface reactions. By tuning gas composition, plasma power, and chamber pressure, the etching rate can be controlled over a wide range. Toros et al. [89] demonstrated that material removal in ICP-RIE fundamentally originates from the synergistic interaction between physical ion bombardment and chemical reactions involving active radicals. Adjusting the ratios of gases such as O_2_, Ar, or Cl_2_ along with the radio frequency power allows for wide-range control extending from low-damage slow etching to high-efficiency deep etching. Typical etching rates under these conditions span approximately 10 to 700 nm/min. Liu et al. [90] utilized self-assembled polystyrene (PS) microsphere masks to achieve the wafer-scale fabrication of diamond microlens arrays. Their research indicates that optimizing the O_2_/Ar flow ratio facilitates the high-fidelity transfer of the mask morphology onto the diamond surface while maintaining surface roughness at the nanometer scale. These results highlight the distinct advantages of ICP-RIE for large-area uniform processing and the manufacturing of periodic array structures. Xie et al. [91] reported that when the incident ion energy in ICP-RIE is reduced to near the chemical reaction energy barrier, an unusual crystal-facet-dependent etching behavior can be activated. This mechanism overcomes the morphological limitations of conventional dry etching, enabling highly selective etching along specific crystallographic planes of diamond. This results in atomically smooth sidewalls inclined at approximately 25° relative to the vertical direction and the formation of nanostructures with semi-apex angles up to 21°, providing a new crystallographic pathway for damage-free shaping of complex optical surfaces. Chen et al. [92] employed antimony-rich inorganic phase-change materials as thermally responsive etch masks, achieving strong in situ pattern protection in Cl_2_/O_2_ plasma. Experimental results showed that the etch selectivity of chromium (Cr) masks was significantly improved to 6:1, enabling deep pattern transfer with a minimum feature size of 100 nm. This coupled advance in reaction kinetics and mask durability provides a solid foundation for large-area, high-aspect-ratio plasma etching of diamond structures. Figure 8 summarizes the development mechanisms and key processing pathways of diamond plasma etching. Based on existing research, it synthesizes the roles of ion bombardment, chemical reactions, mask evolution, and morphology control mechanisms for deep etching in ICP-RIE processes.

Despite these advances, the exceptional chemical inertness of diamond still poses two major engineering challenges for RIE-based fabrication of high-aspect-ratio parabolic deep structures: (1) exceptionally low etch selectivity between diamond and conventional resist materials; and (2) morphology degradation during deep etching. Zhang et al. [93] reported that when conventional photoresists are used directly as masks, the poor etch selectivity results in extremely shallow lens structures. To address premature mask erosion, a “3D double-layer mask” strategy was proposed, in which a single-crystal silicon intermediate layer is introduced. Exploiting the high selectivity between diamond and silicon, high-aspect-ratio micro-lenses with improved surface smoothness and increased height were successfully fabricated. Hicks et al. [94] investigated diamond etching depths exceeding 10 μm and found that sputter redeposition from metal masks (e.g., aluminum) in plasma can induce severe micro-masking effects, leading to extensive grass-like defects. They demonstrated that cyclic Ar/Cl_2_ cleaning steps can effectively remove redeposited mask residues in situ, and that optimizing the Ar/O_2_ mixing ratio at a working pressure of 15 mTorr enables near-micro-mask-free deep etching. Ultimately, high-quality structures with depths of 10.6 μm and surface roughness as low as 0.47 nm were achieved. In summary, innovations in mask engineering and precise optimization of etching parameters remain the central challenges in the ICP-RIE fabrication of high-quality deep diamond refractive lenses.

### 3.4. Hybrid Fabrication Methods

Single fabrication techniques often struggle to achieve an optimal balance among processing efficiency, surface figure accuracy, and lattice integrity. The current fabrication of high-energy diamond X-ray refractive lenses is therefore evolving toward hybrid and composite manufacturing strategies. The core concept of composite processing lies in leveraging the complementary advantages of different techniques: one process is first used for efficient bulk or near-net-shape fabrication, followed by secondary processes for precision finishing or damage repair [95].

The strategy of overcoming single-process limitations through “multi-field synergy” has demonstrated strong interdisciplinary applicability across a wide range of hard-to-machine materials. Qi et al. [96] investigated extremely hard transparent optical materials and first employed femtosecond laser processing for efficient pre-modification and rapid three-dimensional bulk structuring, which was then directly followed by single-step ion beam etching. In this approach, ion beams act as a “nanoscale polishing tool,” effectively removing laser-induced surface roughness and subsurface damage. Results showed that this modification–etching hybrid process enabled the integration of as many as 27,000 cross-scale microlens arrays onto a macroscopic lens surface with a diameter of only 2.5 mm, breaking the inherent trade-off between large-scale shaping and microscale fidelity in single-process fabrication. Conventional infrared femtosecond laser processing of diamond typically induces a relatively large heat-affected zone and leads to the formation of highly stable carbonaceous deposits. Gao et al. [97] demonstrated a hybrid process combining ultraviolet femtosecond laser machining with hot mixed-acid chemical treatment. The shorter wavelength UV laser, with its higher single-photon energy, enables a reduced diffraction-limited spot size and more “cold ablation”-like material removal. Subsequent chemical treatment in a heated mixed-acid solution effectively removed graphitized clusters and amorphous carbon residues from the surface. Experimental results showed that the surface roughness of laser-processed regions decreased significantly from 37.4 nm to 9.4 nm, while the baseline roughness of unprocessed regions was further reduced to the sub-nanometer level of 0.64 nm.

Hybrid fabrication strategies are increasingly emerging as a highly promising route for high-energy diamond X-ray refractive lenses. To provide a clear overview of the overall performance of the aforementioned fabrication techniques, Table 3 compares the key processing metrics. Femtosecond laser processing is particularly well suited for rapid structural prototyping, FIB machining is effective for final-stage nanoscale profile correction, and RIE-based techniques offer clear advantages in batch fabrication and surface uniformity. The rational integration of these complementary approaches is expected to play a central role in achieving both scalable manufacturing and diffraction-limited optical performance in the future.

## 4. Optical Quality, Crystal Integrity and Thermal Stability

Although micro- and nanofabrication techniques such as femtosecond laser processing, FIB machining, and RIE have enabled the realization of three-dimensional diamond X-ray refractive lens structures at the macroscopic scale [69,99], the optical performance in high-energy synchrotron radiation and XFEL applications is not determined solely by the accuracy of the global surface figure. It is also highly dependent on surface microroughness, the intrinsic crystalline quality of the material, and thermal stability under extreme heat loads [22,102]. Micrometer-scale form errors, nanometer-scale surface roughness, and microscopic defects within the crystal lattice [32,102,103] can all induce phase distortions and parasitic scattering, severely degrading the wavefront quality and spatial coherence of the beam [104]. As a result, post-fabrication performance optimization and bulk defect control have become key research priorities in the field of diamond X-ray optics.

### 4.1. Surface Roughness

**Figure 9:**
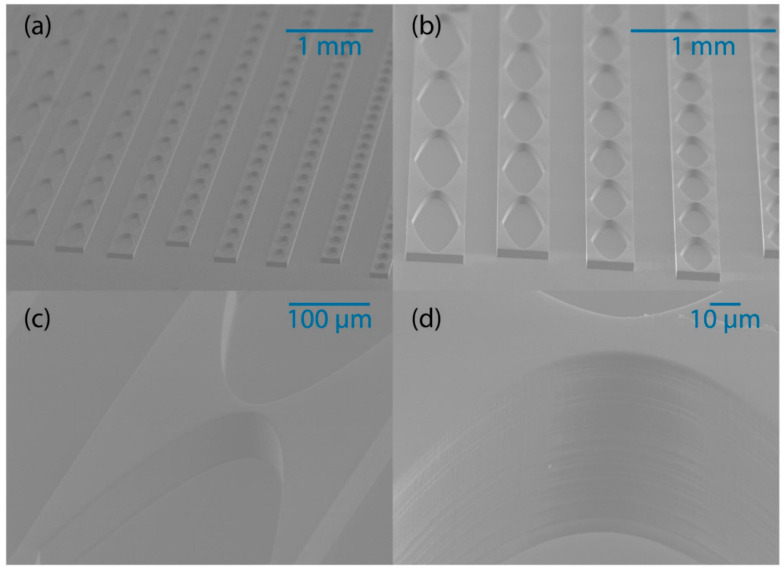
Tilted scanning electron microscopy images [105]. (**a**) low-magnification view showing the apparent absence of extraneous structures around the lenses; (**b**) further magnified view confirming the clean structure layout; (**c**) high-magnification image demonstrating the remarkable form fidelity of the silicon-diamond transfer molding process; (**d**) detailed view where the scalloped surface deriving from the silicon etch is observed.

X-ray wavelengths typically lie in the sub-nanometer to nanometer regime, making X-ray optical components extremely sensitive to surface microtopography. Even when the overall lens geometry is precisely defined, surface roughness can still induce small-angle scattering, focal spot broadening, and degradation of wavefront fidelity. Studies have shown that when the root-mean-square surface roughness exceeds a few nanometers, a pronounced small-angle X-ray scattering background emerges at the interface. The scattering intensity is directly related to the surface power spectral density (PSD) and increases with higher spatial frequencies [75]. To address the challenge of ultra-precision polishing in deep diamond microstructures due to its ultrahigh hardness, Fox et al. [105], as shown in Figure 9, proposed a template-transfer “reverse molding” approach. In this process, a high-smoothness negative template is first fabricated on a silicon wafer using electron-beam lithography and deep reactive ion etching. Diamond is then conformally grown within the template via microwave plasma chemical vapor deposition, followed by selective removal of the silicon substrate through chemical dissolution to release the final optic. This strategy enables the diamond structures to inherit the sub-nanometer surface smoothness of silicon, effectively eliminating thermally induced graphitization and morphology distortions associated with subtractive processing. Without any post-polishing, the method significantly suppresses scattering background and ultimately achieves high-quality focal spots on the order of 2–4 μm. Based on a surface scattering model, Spiga et al. [106] demonstrated that micro-roughness on X-ray mirror surfaces induces scattering in the physical optics regime and constitutes the dominant factor limiting imaging performance in hard X-ray telescopes. Their analysis indicated that, to meet the design targets of the SIMBOL-X mission—namely a half-energy width of 15 arcsec at 1 keV and below 20 arcsec at 30 keV—the surface PSD must be strictly controlled, with particular suppression of roughness components at spatial wavelengths shorter than 300 μm. Calculations further showed that, even when a 15 arcsec figure error is assumed, the scattering-induced contribution to half-energy width increases significantly with photon energy, especially at larger incidence angles where degradation in imaging performance becomes more pronounced. The study provided quantitative PSD-based tolerance criteria and demonstrated that conventional nickel electroforming processes, without further optimization, are insufficient for high-energy X-ray applications. Therefore, controlling sidewall roughness within the sub-nanometer to nanometer range during micro/nanofabrication is a critical requirement for ensuring high focusing efficiency and wavefront quality in high-energy X-ray optics.

At the level of chemical defect healing, the primary objective is to remove the damaged layer and uniformly reduce microroughness. High-temperature selective thermal oxidation can effectively strip the graphitized “dead layer” left by laser rough machining. Based on the higher surface free energy of sp^2^-bonded carbon and sharp nanoscale protrusions, these regions preferentially undergo oxidation and volatilization over the sp^3^-bonded diamond bulk in an oxygen-rich environment at 700–800 °C. Experimental results indicate that this process can non-destructively remove an approximately 50 nm thick laser-induced amorphous layer, restoring the high-energy transmission efficiency of the device to more than 98% of its theoretical value [107,108]. Plasma-assisted polishing and isotropic chemical etching are also effective approaches for global surface smoothing [89,109]. In high-temperature oxygen- or hydrogen-containing plasma environments, highly reactive radicals preferentially react with nanoscale asperities through oxidation–reduction processes, leading to their volatilization [110]. This chemically driven material removal process enables a gradual reduction in surface roughness toward the sub-nanometer regime (~0.5 nm) without compromising the macroscopic figure of the optic [111].

From the perspective of physical surface correction, the focus shifts to local geometric compensation and damage-free ultra-precision polishing. For early-stage fabrication errors that induce severe wavefront distortions, multi-axis nanometer-precision FIB systems are commonly employed for localized deterministic material removal, allowing the surface to approach an ideal diffraction-limited figure [112]. The introduction of gas cluster ion beam smoothing into diamond X-ray optics fabrication has further overcome the intrinsic limitations of conventional monoatomic ion beams, which often introduce subsurface lattice damage. Gas cluster ion beam utilizes clusters containing thousands of atoms to bombard the surface, resulting in extremely low per-atom kinetic energy and strong lateral sputtering effects. This mechanism enables efficient and precise leveling of nanoscale asperities while maintaining a damage-free subsurface, reducing the root-mean-square surface roughness of diamond from approximately 15 nm to the sub-nanometer range of 0.3–0.4 nm [113,114].

### 4.2. Crystal Quality

Modern synchrotron radiation sources and XFEL exhibit extremely high brightness and spatial coherence, which require that the wavefront phase of the beam remains highly uniform as it propagates through refractive optics [22]. Owing to its excellent thermal and mechanical properties, as well as its ability to preserve wavefront quality under high-power X-ray irradiation, diamond has emerged as a highly promising material for such applications. The coherence-preserving performance of diamond optical components is still strongly influenced by their internal microstructure. CVD diamond used in optical applications is generally classified into polycrystalline diamond (PCD) and SCD, which exhibit significant differences in microstructure and optical performance. CVD diamond demonstrates favorable thermal and optical properties, enabling high transparency and low thermal distortion under ultra-high-power irradiation conditions [115]. With the continued development of CVD techniques, the fabrication of large-area, high-purity synthetic diamond has become increasingly mature, laying a solid foundation for its application in advanced high-energy optical systems [116]. The extremely low impurity content of high-quality single-crystal CVD diamond results in exceptionally high carrier mobility at room temperature (electron mobility about 4500 cm^2^/Vs and hole mobility about 3800 cm^2^/Vs), further confirming its ultra-high crystal purity [117].

**Figure 10:**
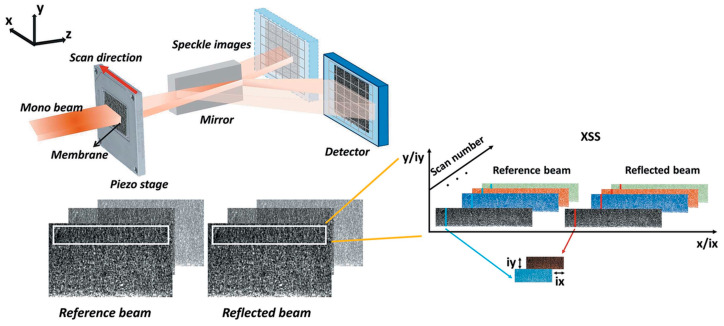
Experimental setup for two-dimensional measurements of X-ray mirrors and data processing workflow [118].

While large-area growth and relatively lower manufacturing cost are key advantages, PCD inherently suffers from microstructural limitations. This material contains a high density of grain boundaries and randomly oriented crystallites, introducing structural disorder and anisotropy at the microscale [22]. These grain boundaries and defects lead to additional X-ray scattering and wavefront distortion, thereby degrading transmitted beam quality. To quantify wavefront errors induced by such defects, Hu et al. [118], as shown in Figure 10, developed a near-field speckle-based two-dimensional scanning metrology technique, enabling ultra-precise measurement of slope errors below 100 nrad on X-ray mirror surfaces and achieving sub-pixel displacement tracking at the detector plane. Achieving ultra-smooth surfaces on polycrystalline diamond remains highly challenging. Liang et al. [119] revealed that during high-speed dynamic friction polishing, the surface roughness of PCD can be reduced from 29.5 nm to 0.9 nm over a 100 μm scale, while the intragranular roughness can reach as low as 0.1–0.2 nm. However, differences in frictional and chemical removal behavior between grain interiors and grain boundaries lead to non-uniform material removal, which remains the key barrier preventing further reduction into the sub-nanometer regime. Consequently, speckle-like intensity fluctuations associated with phase perturbations introduced by optical elements may appear in transmitted wavefronts [22]. For experiments relying on highly coherent X-ray beams, such as coherent diffraction imaging and X-ray photon correlation spectroscopy, these effects are particularly detrimental, as maintaining wavefront coherence is critical [105].

High-quality SCD, owing to its excellent thermal conductivity, superior mechanical properties, and low absorption for hard X-rays, is widely regarded as one of the most suitable materials for high-energy coherent X-ray optics. However, current CVD homoepitaxial growth techniques still face significant challenges in fully eliminating crystalline defects during synthesis. It has been shown that, unlike high-pressure high-temperature diamond in which defects primarily originate from the seed–substrate interface, defects in CVD diamond are mainly manifested as threading dislocations propagating perpendicular to the growth direction [120]. Masuya et al. [121] used synchrotron X-ray topography to confirm a direct correlation between etch pit morphologies on the (111) crystal plane and underlying threading dislocations, providing an effective non-destructive approach for mapping defect distributions. To actively control such defects, Issaoui et al. [122] proposed a “dopant engineering” strategy and demonstrated that appropriate boron–nitrogen co-doping can modify the local lattice stress field, promoting the bending or annihilation of threading dislocations during growth. This approach effectively reduces the dislocation density within selected high-quality regions to a relatively low level. These defects introduce local lattice strain and structural inhomogeneity within the crystal. In X-ray topography and diffraction imaging, dislocations typically appear as dark contrast lines, whereas stacking faults manifest as streak-like distortions in diffraction patterns. The strain fields associated with these defects can significantly degrade the optical quality required for coherent X-ray applications.

During the CVD growth process, it is therefore essential to suppress defect propagation by optimizing plasma parameters and strictly controlling gas purity. Friel et al. [123] pointed out that subsurface damage and microscopic defects on the substrate surface are the primary sources of threading dislocations in the newly grown CVD layer. Consequently, eliminating mechanically induced damage through ultra-precision substrate polishing is critical for achieving stable epitaxial growth of SCD with ultra-low dislocation density. Synchrotron-based X-ray topography is commonly employed to identify regions with minimal birefringence and ultra-low defect density, which can then be selected as “optical-grade” single-crystal areas for the fabrication of diamond refractive lens arrays. A comparison of the properties of different CVD diamond materials is provided in Table 4.

### 4.3. Thermal Stability

Under the ultrahigh photon flux of high-brightness synchrotron radiation sources and XFEL, optical components are subjected to severe thermal loading, making thermal management a key factor governing long-term optical stability. SCD, owing to its exceptional room-temperature thermal conductivity, low thermal expansion coefficient, and superior radiation resistance, offers clear advantages under such extreme conditions and has been established as an ideal material for next-generation two-dimensional X-ray refractive lenses [129].

The real thermal response of SCD under ultra-high repetition rates and ultrahigh pulse energies remains highly complex. Under MHz-repetition XFEL irradiation with millijoule-level pulses, several effects may become significant, including transient thermal deformation, non-diffusive phonon transport deviating from Fourier’s law at the micro- and nanoscale [130], and thermally induced wavefront distortions under kilowatt-level average power pumping [131]. Cryogenic liquid-nitrogen cooling combined with second-order compensation techniques is required to constrain transient thermal deformation to a root-mean-square level below 15 pm [132]. Under full-beam exposure conditions of XFEL pulse trains (e.g., 17.5 keV, 1 mJ, 10 kHz), corresponding to fluences up to 10 J/cm^2^, the peak temperature of optimized multilens systems can still approach 300 °C [133].

**Figure 11:**
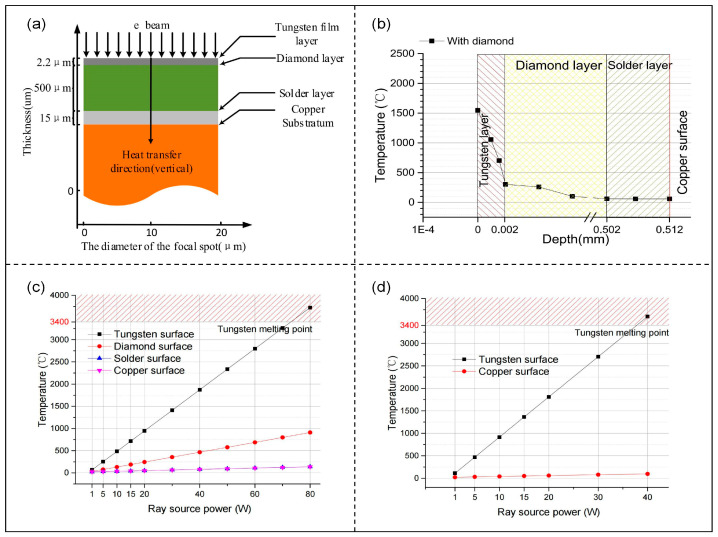
Thermal dissipation models and power limit comparisons for different X-ray anodes [134]. (**a**) Schematic of internal heat transfer directions within the focal spot region; (**b**) vertical temperature gradient within the diamond composite anode; (**c**) power-dependent maximum temperatures for the diamond composite anode with a power limit of 73 W versus (**d**) the conventional tungsten anode with a power limit of 37.9 W.

In practical systems, the primary thermal bottleneck arises from the thermal contact resistance at the interface between the diamond lens and the external cooling substrate. Even though diamond exhibits excellent intrinsic thermal conductivity, inefficient interfacial heat transfer can still lead to localized heat accumulation and significant temperature gradients, which in turn degrade wavefront coherence. Recent research has therefore shifted from isolated material-level considerations toward system-level interface engineering and thermal packaging strategies to enhance thermomechanical stability [32,134]. Table 5 summarizes the primary thermal management and packaging strategies for diamond-based X-ray optics under high-heat-load conditions. Optimizing interfacial heat transfer while simultaneously managing phonon scattering and contact resistance is essential to fully exploit the potential of diamond-based optics. As shown in Figure 11, Li et al. [134] demonstrated that integrating a “diamond thermal buffer layer” within an anode target significantly enhances lateral heat spreading, doubling the operational power limit compared to conventional metallic targets. Diamond–metal composite substrates have thus emerged as a highly promising strategy. Li et al. [135] successfully fabricated a diamond–copper composite substrate with a thermal conductivity of 807 W/(m·K) by combining controlled metallization, engineered carbide interlayers, and active metal brazing. This approach overcomes both weak interfacial bonding and strong phonon scattering at diamond–metal interfaces. Compared with conventional copper heat sinks, the composite structure reduces overall thermal resistance by 39% and lowers the peak operating temperature by 12.3 °C under identical high-power conditions. In practical packaging, additional Ti metallization via vacuum evaporation and tailored brazing alloys can be employed, followed by graded heating and cooling under vacuum conditions to effectively mitigate residual thermal stresses.

Another important development direction is the integration of microchannel cooling technologies. Microchannels offer a very high effective convective heat transfer area and are therefore highly advantageous for high-power thermal management. Although the direct fabrication of deep, high-aspect-ratio microchannels in ultra-hard diamond substrates remains challenging, progress has been achieved through hybrid laser-based strategies. Cui et al. [136] developed a precision ultraviolet (355 nm) nanosecond laser machining approach based on a multi-feed strategy. By employing a spiral scanning path for dynamic energy distribution, they successfully fabricated straight microchannels in polycrystalline diamond with a depth of up to 800 μm, an aspect ratio of 4:1 (up to 16.7 in optimized cases), and a sidewall taper angle of only 0.9°. The process achieved a material removal rate of 17.3 × 10^−3^ mm^3^/s, while maintaining the bottom roughness at approximately 186 nm. This work provides a practical pathway for integrating efficient thermal management architectures directly into diamond-based optical components.

## 5. Applications and Integration

With the advancement of micro- and nanofabrication processes, improved control over crystalline defects, and the maturation of system-level thermal packaging technologies, diamond X-ray refractive lenses have gradually moved from laboratory-scale theoretical studies and proof-of-concept demonstrations toward practical engineering applications. High-quality diamond CRL arrays have now become key optical components supporting next-generation high-energy X-ray sources [31,71].

Since the first introduction of the CRL concept by Snigirev et al. [26], hard X-ray focusing technology has undergone a paradigm shift. Early work demonstrated that linear arrays of cylindrical holes fabricated in low-absorption materials could successfully focus 14 keV hard X-rays down to an 8 μm line width, challenging the long-standing assumption that X-rays cannot be efficiently focused by refraction. Continuous progress in refractive optics has further pushed spatial resolution limits. Schroer et al. [137] developed hard X-ray nanoprobes based on microfabricated parabolic lenses and achieved a focused spot size of 47.55 nm^2^ at 21 keV, confirming the strong potential of CRL for nanoscale imaging applications.

Driven by increasingly stringent requirements on thermal stability and structural robustness in next-generation high-brilliance sources, diamond has progressively replaced conventional materials as the primary platform for high-performance CRL fabrication. To overcome the extreme machining difficulty associated with SCD, Antipov et al. [31] combined high-precision laser ablation with post-polishing processes to fabricate parabolic lenses and demonstrated their first successful two-dimensional (2D) micron-scale focusing performance on a synchrotron beamline. This work established the feasibility of SCD as an advanced optical material and marked a significant step forward in the fabrication of complex high-precision optical geometries.

To further improve form fidelity and enable array-based manufacturing, Lyubomirskiy et al. [71] introduced planar micro- and nanofabrication strategies, integrating photolithography with deep RIE. Using this approach, diamond lens arrays were fabricated in a batch process and achieved a focal spot size of 150 nm under hard X-ray illumination, providing a scalable route toward next-generation high-resolution X-ray microscopy. A comprehensive overview of recent progress is summarized in Table 6, which systematically compiles state-of-the-art microfabrication processes and the corresponding ultimate optical performance achieved by leading research groups. The data indicate that both two-dimensional parabolic lenses and phase-modulated refractive structures are steadily approaching the theoretical focusing limit of hard X-rays.

Accurate theoretical optical models play a critical role in system-level integration and beamline implementation of diamond refractive lenses, providing essential guidance for design optimization. Kohn et al. [138] systematically investigated the “effective aperture” of CRL by incorporating photon absorption and Compton scattering effects, and derived a more rigorous analytical formulation. This model corrects previous deviations in the evaluation of low-atomic-number materials such as diamond, enabling more accurate predictions of transmission efficiency, diffraction-limited resolution, and focusing gain. Coupled with advances in theoretical design and micro/nanofabrication technologies, diamond refractive lenses are steadily expanding their application scope in cutting-edge high-energy X-ray science.

## 6. Challenges and Future Perspectives

### 6.1. Challenges

Despite significant advances in diamond micro- and nanofabrication and system-level integration, as well as the successful demonstration of high-quality diamond refractive lenses in state-of-the-art high-energy beamlines, several critical bottlenecks remain in material synthesis and device fabrication, limiting the full exploitation of their potential in next-generation high-energy X-ray sources [32,71,139,140]. To clearly organize the key obstacles in current technological development, Table 7 systematically summarizes the major challenges in diamond X-ray lens fabrication, covering both crystal growth and micro/nanomanufacturing stages, together with the corresponding impacts on final optical performance.

Diamond, with its exceptionally high refractive index decrement and extremely low absorption cross section, is regarded as an ideal material for CRL operating in high-energy X-ray regimes. The transition from laboratory-scale material advantages to industrial implementation remains highly challenging [141]. For X-ray optical components, the key material-side issue lies in balancing large aperture size with high crystal perfection.

Conventional SCD grown via high-pressure high-temperature or microwave plasma-enhanced CVD is typically limited to sizes below 10 × 10 mm^2^, which is insufficient for large-aperture X-ray optical systems. To overcome this limitation, stitched SCD growth has become a mainstream approach. Large-area stitched crystals up to 30 × 30 × 1 mm^3^ have been successfully demonstrated [142], and numerical simulations optimizing flow and thermal fields have enabled the fabrication of 2-inch-scale stitched single crystals [143]. Despite the apparent scalability, such assembled crystals inevitably suffer from reduced physical continuity. The central challenge in stitched growth lies in the healing of interfaces between individual seeds. Studies have shown that even slight angular misalignment and height mismatch between seeds significantly affect surface morphology at the interface [144]. During growth, step bunching readily occurs at the boundary region, and polycrystalline inclusions may be induced, leading to optical inhomogeneity [141]. To accelerate seam healing, nitrogen is often introduced into the MPCVD environment to enhance lateral growth rates of the substrate. Although this approach reduces fabrication time, it introduces potential impurity-related risks [145]. For X-ray optics, localized stress concentration can result in wavefront distortion. Confocal Raman mapping has revealed pronounced stress peaks at stitching boundaries, frequently accompanied by the accumulation of non-diamond sp^2^ carbon phases [146]. Dislocations in CVD diamond are typically inherited from the initial seed crystals and propagate along the growth direction during epitaxy [120]. The uniformity of seed thickness and the pre-treatment process (e.g., plasma etching) are therefore critical to the final crystal quality. Experimental results indicate that thickness variations between seeds are a primary cause of high dislocation density and stress accumulation at stitched interfaces, requiring strict pre-growth screening and in situ etching optimization [147].

While stitching technology addresses the dimensional requirement of diamond lenses in terms of “quantity,” significant material challenges remain in terms of “quality,” particularly in suppressing interfacial stress fields, dislocation evolution, and non-diamond phase inclusions, which are the main material bottlenecks for high-performance X-ray refractive lens fabrication.

Diamond is the hardest known material in nature, and conventional mechanical machining methods are unable to achieve nanometer-scale surface roughness and micrometer-level form accuracy. Current mainstream fabrication techniques for X-ray lenses are therefore undergoing a transition from macroscopic structural shaping toward micro- and nanoscale precision control.

Laser processing is one of the fastest approaches for fabricating diamond lenses. Femtosecond laser ablation has been successfully used to fabricate two-dimensional focusing lenses with hyperbolic profiles on SCD substrates [31]. Although this technique demonstrates the stability of diamond optics under high-flux synchrotron radiation, the thermal-affected zone and surface graphitization induced during laser processing can increase surface roughness. Geometric deviations introduced by laser ablation directly degrade the X-ray wavefront quality. While deep structures can be achieved, the elimination of ablation pits and microcracks through post-processing remains a key challenge. To achieve higher surface quality and preserve X-ray coherence, plasma-based dry etching techniques have been explored. Oxygen plasma is commonly used for diamond etching; however, most mask materials, such as photoresists, are rapidly consumed in oxygen-rich environments. Yang et al. [148] proposed a SiO_2_/Cr bilayer mask strategy, which improves the etch selectivity through a dual-layer architecture. Experimental results show that this hybrid mask significantly reduces edge roughness and increases the diamond-to-mask etch selectivity to several times that of conventional single-layer masks, enabling the fabrication of micro-optical structures with higher aspect ratios. Meng et al. [149] reported that lenses fabricated via inductively coupled plasma etching exhibit superior sidewall morphology compared with laser-processed structures, which is crucial for maintaining interference fringe contrast in coherent X-ray applications. FIB processing provides exceptional fabrication flexibility and nanometer-scale positioning accuracy. Marseglia et al. [150] used FIB milling to fabricate solid immersion lenses on diamond surfaces, achieving approximately a tenfold enhancement in fluorescence collection efficiency. Although FIB enables extremely high-precision lens shaping, its processing efficiency is very low, limiting its application to single micro-scale lenses. For CRL arrays composed of tens of lens elements, FIB is not suitable for large-area or scalable fabrication.

### 6.2. Summary and Outlook

This review systematically summarizes recent advances in the fabrication and optimization of diamond-based X-ray refractive lenses for high-energy applications. In the context of fourth-generation synchrotron radiation sources and XFEL, conventional X-ray optical materials are approaching their physical and thermodynamic limits under severe thermal loads and intense radiation environments. Diamond, with its exceptional thermal conductivity, outstanding mechanical strength, and ultra-low X-ray absorption, has emerged as a key material for next-generation extreme X-ray optics. To address the fabrication challenges associated with ultra-hard materials, a range of advanced manufacturing approaches has been developed, including femtosecond laser ablation, FIB precision machining, and RIE-based large-scale pattern transfer. Significant progress has also been achieved in surface smoothing, crystalline defect control, and system-level thermal packaging.

Future development of diamond X-ray optics is expected to focus on two main directions:

Deep integration of hybrid micro- and nanofabrication processes: Future fabrication paradigms will shift away from single-step approaches toward fully integrated workflows that combine high-efficiency material removal via ultrafast lasers, large-area pattern transfer via RIE, and atomic-scale surface refinement through damage-free plasma polishing. Such multi-field hybrid processes are expected to overcome the long-standing trade-off between fabrication efficiency and optical precision.

Exploration of active and adaptive diamond optical devices: With continuously increasing coherence of next-generation light sources, static lens geometries are becoming insufficient to fully compensate for dynamically evolving wavefront distortions induced by high heat flux in beamline environments. Inspired by recent advances in active and adaptive X-ray optics, future designs may integrate piezoelectric micro-actuators or microscale thermal gradient control arrays to enable dynamic curvature tuning and real-time wavefront correction of diamond lenses.

With the continuous evolution of large-scale single-crystal growth technology and extreme micro- and nano-fabrication processes, diamond X-ray refractive lenses are poised to play an irreplaceable, pivotal role in advancing the frontiers of hard X-ray nano-focusing, capturing ultrafast material dynamics, and enabling high-resolution imaging in the life sciences.

## Figures and Tables

**Table 1:** Evolution of optical system requirements across different development stages of X-ray sources. (The unit of brightness is photons·s^−1^·mm^−2^·mrad^−2^·(0.1% bandwidth)^−1^ [1]).

Light Source Stage	Average Brightness	Core Requirements for Optical Systems	Key Challenges
GSR	~10^12^	Basic transport and focusing	Geometric precision
ALS	~10^20^	High-resolution imaging and stable focusing	Wavefront error control
ALS-U	~10^22^	Nanoscale wavefront control	Thermal drift
SASE-XFEL	~10^24^	Ultrafast imaging and coherent diffraction	Thermal shock and radiation damage
XFEL-O	~10^28^	Wavefront fidelity and low noise	Long-term stability

**Table 2:** Comparison of the properties of X-ray optical materials.

Material	Thermal Conductivity (W/m·K)	Coefficient of Thermal Expansion (10^−6^/K)	Absorption Coefficient 10 keV (cm^−1^)	Core Limitations
Single-crystal diamond	2250 [50]	~1.1 [51]	~8.33 [52]	Challenges in micro-nanofabrication, high cost for large-scale growth
Metallic beryllium	200 [53]	~11.3 [54]	~1.2 [52]	Significant thermal distortion, toxicity, anisotropy
Single-crystal silicon	140 [50]	~2.6 [55]	78.94 [52]	Pronounced absorption in high-energy regions

**Table 3:** Comparison of key technical parameters for micro- and nano-machining of diamond.

Machining Technique	Precision	Removal Rate	Advantages	Limitations
Femtosecond laser ablation	Sub-micrometer to micrometer	3.64 × 10^5^ μm^3^/s at 56.6 J/cm^2^, 1 kHz, 1 mm/s [98]	High throughput, versatile for 3D micro-structuring [99].	Significant surface roughness.
FIB	Nanoscale	Ga^+^ 0.4–0.6 μm^3^/nC [100]	Exceptional precision; superior surface finish.	Low throughput, prone to ion-induced damage.
RIE	Nanoscale to sub-micrometer	50–100 nm/min [101]	Good surface quality and uniformity.	Requires lithographic masking processes.

**Table 4:** Comparison of microstructural characteristics and performance of different CVD diamond materials in X-ray optics.

Diamond Type	Typical Dimensions (mm^2^)	Core Internal Crystalline Defects	Thermal Conductivity W/(m·K)	Typical X-Ray Wavefront Distortion	Application Scenarios
SCD	>10 × 10	Threading dislocations [120]	~2200 [124]	Bragg-plane slope error ~0.15–0.2 μrad/mm^2^ [125]	Nanofocusing lenses, monochromators
PCD	Inch-scale	Grain boundaries and orientation mismatch	400–2200 [126]	Significant scattering and coherence degradation [44]	White beam windows, high-heat-load windows
Mosaic SCD	30×30 to 2 inches	Seam stress and height mismatch	~2400 [127]	Localized phase perturbation and reflection broadening [128]	Large-aperture XFEL optics

**Table 5:** Thermal management and packaging strategies for diamond optical elements under extreme heat loads.

Strategy	Operating Conditions	Key Performance Indicators	Advantages	Challenges	References
Cryogenic cooling and second-order compensation	XFEL, 1 MHz	Thermal distortion RMS < 15 pm.	Good wavefront stability	System complexity	[132]
Flexible thermal interface materials	High-heat-flux synchrotron radiation	Reduction of residual stress	Buffering thermal mismatch	Insufficient long-term stability	[134]
Diamond–copper composite substrates	High heat flux density	Thermal resistance reduced by 39%; ΔT decreased by 12.3 °C	Enhanced heat dissipation	Difficulties in interfacial bonding	[135]
Direct microchannel heat sinks	Aspect ratio 4:1–16.7	Surface Sa ≈ 186 nm	High heat exchange efficiency	Complex fabrication	[136]

**Table 6:** Frontier applications and performance limits of diamond CRL.

Lens Structure Type	Core Micro-Nano Fabrication Process	Light Source & Energy Range	Focusing & Key Optical Performance Highlights	References
SCD 2D Parabolic Lens	High-precision laser ablation and mechanical polishing	Synchrotron hard X-rays	Maintained wavefront fidelity under high heat load; achieved micron-scale 2D focusing.	[31]
PCD CRL	Femtosecond laser ablation	Synchrotron (9.25 keV)	First verification of the efficacy of laser-fabricated lenses, achieved effective focusing at 97 cm.	[66]
2D Phase-shifting Lens	FsLDW	Synchrotron (14 keV)	Achieved an ultra-small focal spot of 52 nm × 51 nm; significantly reduced absorption losses.	[69]
Planar Nano-focusing Lens	“Chip-scale” surface milling and figuring via FIB	Synchrotron hard X-rays	Eliminate residual wavefront aberrations to achieve a focal spot of approximately 50 × 60 nm^2^.	[70]
2D Nano-focusing CRL Array	Planar micro-fabrication	Synchrotron hard X-rays	Successfully achieved ultra-high-resolution focal spots (<200 nm), demonstrated potential for array integration.	[71]
High-smoothness Polycrystalline Microlens	“Molding-transfer”: CVD growth on patterned Si-wafer molds	Synchrotron hard X-rays	Obtained 2–4 μm focal spots without post-polishing; significantly suppressed small-angle scattering.	[105]

**Table 7:** Current challenges in diamond X-ray lens research.

Critical Challenges	Underlying Causes	Impact on Optical Performance	Existing Solutions	Future Research Directions
Surface roughness	Laser ablation	Enhanced scattering	Plasma polishing	Atomic-scale surface finishing
Subsurface damage	Ion bombardment	Wavefront distortion	Low-energy Ion polishing	Damage-free fabrication technologies
Figure error	Fabrication precision	Focal spot deviation	FIB figuring	Adaptive machining
Thermal stability	High-power beams	Optical drift	Cooling structure Design	Advanced materials

## Data Availability

Not applicable.

## Abbreviations

The following abbreviations are used in this manuscript:

CRL	Compound refractive lenses
CVD	Chemical vapor deposition
FIB	Focused Ion beam
FsLDW	Femtosecond laser direct writing
ICP-RIE	Inductively coupled plasma reactive ion etching
PCD	Polycrystalline diamond
PSD	Power spectral density
RIE	Reactive ion etching
SCD	Single-crystal diamond
XFEL	X-ray free-electron lasers
